# Blood Serum Stimulates p38-Mediated Proliferation and Changes in Global Gene Expression of Adult Human Cardiac Stem Cells

**DOI:** 10.3390/cells9061472

**Published:** 2020-06-16

**Authors:** Anna L. Höving, Kazuko E. Schmidt, Madlen Merten, Jassin Hamidi, Ann-Katrin Rott, Isabel Faust, Johannes F. W. Greiner, Jan Gummert, Barbara Kaltschmidt, Christian Kaltschmidt, Cornelius Knabbe

**Affiliations:** 1 Department of Cell Biology, University of Bielefeld, 33615 Bielefeld, Germany; kazuko_elena.schmidt1@uni-bielefeld.de (K.E.S.); jhamidi@uni-bielefeld.de (J.H.); ann-katrin1207@t-online.de (A.-K.R.); johannes.greiner@uni-bielefeld.de (J.F.W.G.); 2 Institute for Laboratory- and Transfusion Medicine, Heart and Diabetes Centre NRW, Ruhr University Bochum, 32545 Bad Oeynhausen, Germany; ifaust@hdz-nrw.de (I.F.); cknabbe@hdz-nrw.de (C.K.); 3 AG Molecular Neurobiology, University of Bielefeld, 33615 Bielefeld, Germany; Madlen.merten@uni-bielefeld.de (M.M.); Barbara.Kaltschmidt@uni-bielefeld.de (B.K.); 4 Department of Thoracic and Cardiovascular surgery, Heart and Diabetes Centre NRW, Ruhr-University Bochum, 32545 Bad Oeynhausen, Germany; jgummert@hdz-nrw.de

**Keywords:** blood serum, heart stem cells, p38-MAPK, RNAseq

## Abstract

During aging, senescent cells accumulate in various tissues accompanied by decreased regenerative capacities of quiescent stem cells, resulting in deteriorated organ function and overall degeneration. In this regard, the adult human heart with a generally low regenerative potential is of extreme interest as a target for rejuvenating strategies with blood borne factors that might be able to activate endogenous stem cell populations. Here, we investigated for the first time the effects of human blood plasma and serum on adult human cardiac stem cells (hCSCs) and showed significantly increased proliferation capacities and metabolism accompanied by a significant decrease of senescent cells, demonstrating a beneficial serum-mediated effect that seemed to be independent of age and sex. However, RNA-seq analysis of serum-treated hCSCs revealed profound effects on gene expression depending on the age and sex of the plasma donor. We further successfully identified key pathways that are affected by serum treatment with p38-MAPK playing a regulatory role in protection from senescence and in the promotion of proliferation in a serum-dependent manner. Inhibition of p38-MAPK resulted in a decline of these serum-mediated beneficial effects on hCSCs in terms of decreased proliferation and accelerated senescence. In summary, we provide new insights in the regulatory networks behind serum-mediated protective effects on adult human cardiac stem cells.

## 1. Introduction

Cardiovascular diseases are the major cause of death worldwide and are accompanied by decreased proliferation capacities of resident cardiomyocytes and endothelial cells and a reduced regenerative potential of quiescent cardiac stem cells (CSCs) [1,2]. In addition to cardiovascular diseases, an overall decline in tissue regenerative potential during human ageing is considered a crucial factor for the onset of other age-related diseases such as Alzheimer’s disease, diabetes, chronic obstructive pulmonary disease and atherosclerosis [3]. Within the natural ageing process, regenerative capacities of endogenous stem cell pools decline. This phenomenon is accompanied by the accumulation of senescent cells in various tissues, responsible for deteriorated organ function [4]. Thus, rejuvenating treatment strategies are needed to increase the endogenous regenerative capacities of adult stem cells. Since the loss of regenerative capacities can be detected in nearly all organs of the human body, the question arises of whether this decline is organ-specific. However, with increasing age, patients are often affected by multiple degenerative diseases, which points in the direction that these are maybe triggered by systemic factors. In this regard, several studies have demonstrated a rejuvenating effect of young blood and blood plasma on various adult stem cell populations, while the discussion about the active component of rejuvenating blood plasma or serum and the underlying molecular mechanisms has not yet come to a consensus. As one example, Conboy and colleagues described for the first time the rejuvenation of muscle satellite cells in aged mice that underwent heterochronic parabiosis through reactivation of Notch signaling [5]. Further, in the same experiments the authors also showed enhanced proliferation of hepatocytes, which was due to reduced levels of the chromatin remodeling factor Brm in old heterochronic parabionts [5]. On that topic, emerging evidence on the existence of rejuvenating or pro-aging blood borne factors was provided in the following years, mainly focusing on heterochronic parabiosis experiments. For instance, the Wyss-Coray group investigated the impact of young blood to the aging brain, especially the hippocampus, and demonstrated beneficial effects on the molecular, structural, functional and even cognitive level [6]. Importantly, they were also the first to show that human umbilical cord plasma is able to revitalize hippocampal function in aged mice and hypothesized the protein TIMP2 to be a crucial player in this process [7].

The results of these studies suggest more than one molecular mechanism is activated by young blood serum and responsible for these rejuvenating effects that may act organ or tissue-specifically. However, the translation from mice to the human system seems to be complicated, as publications of convincing results are extremely rare [8]. However, the first clinical trials are currently aiming to treat neurodegenerative diseases with either young plasma transfusions or age-related plasma fractions, so-called chronokines [9]. Next to neurodegeneration, a decline in cardiac function is another significant risk factor during human ageing. Although the adult heart was long ago considered a terminally differentiated organ with low regenerative potential, rare populations of mouse and human cardiac stem cells (CSCs) were found that may contribute to endogenous repair mechanisms [10,11,12]. Most of these CSCs were defined by the expression of the stem cell factor receptor kinase cKit [10,13,14,15,16]. However, a range of other cell surface markers has also been reported. For instance, a population of mouse embryonic Isl1^+^ stem cells was shown to give rise to cardiomyocytes, endothelial cells and smooth muscle cells [17]; additionally, mouse Sca1^+^/CD31^+^/cKit^−^ cardiac stem cells were described to exhibit regenerative capacities [18]. In the human system, Smits and colleagues described the isolation of a Sca1^+^/CD105^+^/CD31^+^ population that differentiates to cardiomyocytes in vitro [12]. Interestingly, a high cardiomyocyte proliferation has been reported during fetal development, in contrast to decreased proliferation capacity in adult hearts [1,19]. Thus, targeting the proliferation potential of adult stem cells seems to be a crucial step to enhancing endogenous repair mechanisms. It is well known that blood plasma or serum is a powerful additive in cell culture to enhance proliferation [20,21,22,23]. Here, most studies focused on the in vitro expansion of human mesenchymal stem cells (MSCs) or hematopoietic stem cells (HSCs) prior to transplantation, highlighting the multiple advantages of human plasma or serum compared with fetal calf serum (FCS) in terms of safety and clinical applicability. However, the effect of human blood plasma on adult human cardiac stem cells and the respective underlying mechanisms still remain unknown.

In the present study, we isolated a Nestin^+^/S100^+^ cell population from the human heart auricle that expresses common cardiac progenitor markers such as CD105, CD31 and Sca1. These cells are able to form cardiospheres after clonal growth and differentiate into cardiomyocyte-like cells in vitro. Moreover, we demonstrate that human blood plasma and blood serum significantly increase proliferation and metabolic activity of human cardiac stem cells. Further, we provide RNA-seq data to investigate the global transcriptome of blood-serum-treated human stem cells and thereby analyze the highly complex regulatory networks that enhance stem cell proliferation. Here, we could successfully identify key pathways that are affected by serum treatment with p38-MAPK and play a regulatory role in protection from senescence and in the promotion of proliferation.

## 2. Materials and Methods

### 2.1. Isolation and Cultivation of Adult Human Cardiac Stem Cells from Heart Auricles

Human heart auricles of left atrial appendages were removed from patients undergoing routine heart surgery after informed and written consent according to local and international guidelines (Declaration of Helsinki). Isolation of human cardiac stem cells (hCSCs) and further experimental procedures were ethically approved by the ethics commission of the medical faculty of the Ruhr University Bochum (approval reference number eP-2016-148). After surgical removal, biopsies were cut into small pieces and washed in PBS (Sigma Aldrich, St-Louis, MO, USA). For initial expansion, the tissue clumps were seeded in gelatin B-coated 10 cm Petri dishes (Sarstedt AG and Co., Nürmbrecht, Germany) with human cardiac stem cell medium (hCSC medium) consisting of DMEM/F-12 medium (Sigma Aldrich), basic fibroblast growth factor (bFGF, 5 ng/mL; Peprotech, Hamburg, Germany), epidermal growth factor (EGF, 10 ng/mL; Peprotech) and 10% fetal calf serum (VWR, Radnor, PA, USA). After reaching confluence, tissue clumps were removed and passaging was performed by treatment with trypsin-EDTA (Sigma Aldrich). For further cultivation, cells were again seeded in gelatin B-coated T-25 cell culture flasks (Sarstedt AG and Co.) in hCSC medium. For clonal analysis, cells were seeded in a 96-well plate (Sarstedt AG and Co.) at a density of 1 cell per well. Single cell dilution was verified by microscopy and medium was changed every two to three days. Sphere forming capacity was tested in low-adhesion culture flasks (Greiner Bio-One, Kremsmünster, Austria) with stem cell medium [24]. In the case of this study, cells from a 77-year old female individual were used.

### 2.2. Immunohistochemistry and Immunocytochemistry

Cryosections of the heart auricle tissue or cultivated cells were fixed for 20 min using 4% paraformaldehyde (PFA), washed and permeabilized in PBS with TritonX-100 (tissue: 0.2%, cells: 0.02%, Applichem, Darmstadt, Germany) and supplemented with 5% goat serum for 30 min. The applied primary antibodies were diluted in PBS as followed: mouse anti-Nestin 1:200 (Millipore, Burlington, MA, USA), rabbit anti-S100B 1:500 (Dako, Glostrup, Denmark), rabbit anti-α-actinin (Cell-Signaling, Danvers, MA, USA), mouse anti Connexin 43 (Millipore). They were applied for 1 h (cells) or for 2 h (sections), both at room temperature (RT). After three washing steps, secondary fluorochrome-conjugated antibodies (Alexa 555 anti-mouse or Alexa 488 anti-rabbit, Invitrogen, Life Technologies GmbH, Carlsbad, CA, USA) were applied for 1 h at RT with a dilution ratio of 1:300. Nuclear staining was realized by incubation with 4,6-diamidin-2-phenylindol (DAPI) (1 μg/mL, Applichem) in PBS for 15 min at RT. Finally, the samples were mounted with Mowiol (self-made). Imaging was performed using a confocal laser scanning microscope (CLSM 780, Carl Zeiss, Oberkochen, Germany) and image processing was executed with ImageJ and CorelDRAW [25] (open source and Corel Corporation).

### 2.3. Flow Cytometry

Cultivated hCSCs were harvested by centrifugation after treatment with trypsin and subsequently stained with PE-coupled anti-CD105, anti-CD117, anti-Sca1 or anti-CD31 antibody (Miltenyi Biotec, Bergisch Gladbach, Germany) according to manufacturer’s guidelines. For isotype controls, hCSCs were stained with PE-coupled IgG1 control antibody or APC-coupled IgG1 control antibody. Analysis was done using Gallios Flow Cytometer (Beckmann Coulter Inc., Brea, CA, USA), while Kaluza Acquisition Software (Beckmann Coulter Inc.) was used for subsequent data acquisition and statistical analysis.

### 2.4. Cardiac Differentiation of hCSCs

Cardiac differentiation of the isolated cells was induced following the protocol described by Smits and colleagues [12]. Briefly, cells were seeded with a density of 10^5^ cells per 6-well in hCSC medium. After 24 h, differentiation was induced with a cardiac differentiation medium consisting of a 1:1-mixture of IMDM (Gibco, Thermo Fisher Scientific, Waltham, MA, USA) and Ham’s F12 nutrient mixture with GlutaMAX-I (Gibco), containing 10% horse serum (Dianova, Hamburg, Germany), 1x MEM nonessential amino acids (Bio Whittaker, Lonza, Basel, Switzerland) and 1x insulin-transferrin-selenium (Gibco). Then, 5 µM 5-azacytidine was added in three consecutive days and differentiation medium was refreshed at day 4. Six days after the start of the differentiation, ascorbic acid (Sigma Aldrich) was added every two days and 1 ng/mL transforming growth factor β (TGF-β) (Peprotech, Hamburg, Germany) was added twice weekly. Medium was refreshed every two to three days. After 28 days, the protein expression was analyzed by immunocytochemical staining for α-actinin as described above. As undifferentiated control, cells were cultured in hCSC medium.

### 2.5. Blood Plasma

Blood plasma samples were collected from routine blood donation service from healthy individual donors. For further comparisons, plasma donors older than 60 years were declared “old,” and donors younger than 20 years were declared “young”. For the isolation of serum from fresh frozen plasma (FFP), 20% CaCl_2_ was added in a ratio of 1:50 and incubated at 4 °C overnight. After centrifugation at 1920 RCF for 20 min, blood serum was harvested from the supernatant. In all assays, blood plasma or serum from three different donors was used as biological replicates within the treatment groups.

### 2.6. p38-MAPK Inhibition

The following p38-MAPK inhibitors were used: BMS-582949 (InvivoChem, Libertyville, IL, USA) and SB239063 (Medchemexpress, Sollentuna, Sweden). BMS-582949 binds to the p38α and induces a less accessible conformation of the activation loop. We further selected SB239063 since it is highly selective (>220-fold selectivity over ERK and JNK1). Inhibitor stock solutions were dissolved in DMSO at a concentration of 10 mM and aliquots were stored at −80 °C. For p38-MAPK inhibition, inhibitors were diluted to 50 µM in the respective assays. DMSO served as control.

### 2.7. Proliferation Assay

For examination of cell proliferation, a determined cell count was seeded in either 6-well TC-plates (Sarstedt AG and Co.) or TC25 cell culture flasks (Sarstedt AG and Co.). The cells were starved for 48 h in serum-free medium containing DMEM-F12 (Sigma-Aldrich), 200 mM L-glutamine (Sigma-Aldrich), 10 mg/mL penicillin/streptomycin (Sigma-Aldrich), 10 ng/mL EGF (Peprotech) and 5 ng/mL FGF-2 (Peprotech) and then treated with 10% of individual blood serum and p38 inhibitors. Medium, blood serum and p38 inhibitors were renewed every two days. The cells were detached using trypsin (Sigma-Aldrich) and cell count was carried out using a Neubauer chamber.

### 2.8. Orangu Cell Viability Assay

A cell viability assay using the Orangu Cell Counting Solution (Cell Guidance Systems, Cambridge, UK) was performed with hCSCs in a 96-well TC-Plate (Sarstedt AG and Co.) in hCSC medium. One thousand cells per treatment and a calibration line of 250, 500, 750, 1000, 1500, 2000, 2500 and 3000 cells were seeded. The cells were treated with 10% blood plasma for two days. For evaluation, the cells were incubated with 10 µL Orangu solution for two hours in the dark. Absorbance at 450 nm was measured using a GloMax microplate reader (Promega, Madison, WI, USA).

### 2.9. Senescence-Associated β-Galactosidase Assay

Activity of Senescence-associated β-Galactosidase was measured according to Debacq-Chainiaux and colleagues [26]. Briefly, cells were washed in PBS (Sigma-Aldrich) and fixed with 4% paraformaldehyde (Sigma-Aldrich) before addition of the staining solution containing 1 mg/mL X-Gal (Carl Roth, Karlsruhe, Germany). Incubation for 18 h at 37°C led to final staining, which could be visualized by phase contrast microscopy.

### 2.10. RNA Isolation and Sequencing

RNA was isolated from cell pellets using the TRI Reagent Protocol for Suspension Cells (Sigma-Aldrich, Darmstadt, Germany) according to the manufacturer’s guidelines. The amount of isolated RNA was determined using a NanoDrop (Thermo Fisher Scientific, Waltham, MA, USA). For storage the samples were kept at −80 °C. Library preparation and sequencing on Illumina Hiseq4000 platform was carried out by Novogene (Beijing, China). After alignment to the reference genome GRCh38 with TopHat v2.0.9, gene expression quantification was performed with HTseq v0.6.1. Raw data are accessible at NCBI Gene Expression Omnibus. Differential gene expression was analyzed using DESeq2 R package (2_1.6.3) and correlation was calculated with the cor.test function. The database DAVID was used for the calculation of overexpressed Gene Ontology (GO)-Terms and pathway analyses [27]. GO terms of differentially expressed genes were determined using the PANTHER classification system [28,29,30] and analysis of Kyoto Encyclopedia of Genes and Genomes (KEGG) pathway enrichment was performed using KOBAS 3.0 [31,32].

### 2.11. ELISA

To measure the protein-contents in terms of MCP1, GDF11 and eotaxin of the plasmas used, the following Kits were used according to manufacturer’s guidelines: Human MCP-1 (CCL2) Mini ABTS ELISA Development Kit (Peprotech), Human Eotaxin (CCL11) Standard ABTS ELISA Development Kit (Peprotech) and Human Growth Differentiation Factor 11 GDF11 ELISA Kit (Novatein Biosciences, Woburn, MA, USA).

## 3. Results

### 3.1. Identification of Nestin^+^/S100B^+^ Cells in the Human Heart Auricle Tissue

**Figure 1:**
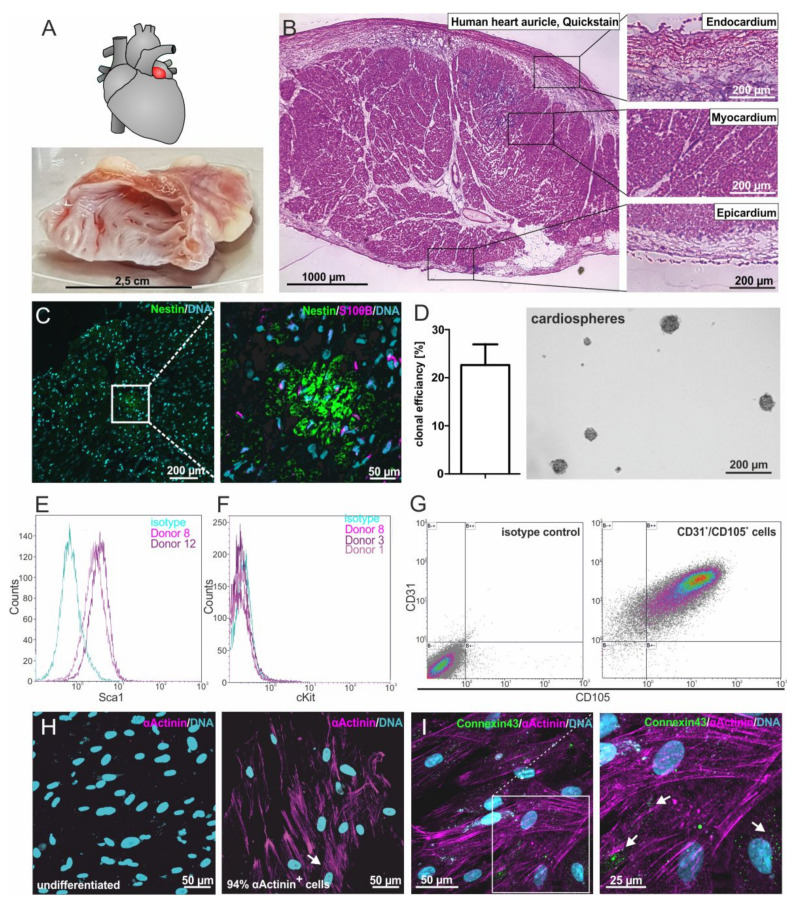
Isolation and characterization of adult human cardiac stem cells (hCSCs). (**A**) Human heart auricles were removed during routine heart surgery. (**B**) Heart auricle tissue consists of the three main layers: endocardium, myocardium and epicardium. (**C**) Nestin^+^ and S100^+^ cells can be found in the myocardium of the human heart auricle. (**D**) Isolated cells spontaneously form cardiospheres and possess the ability for self-renewal with a clonal efficiency of 22.7%. (**E**) Cultured cells express the cardiac stem cell marker Sca1. (**F**) Cultured cells do not express the stem cell marker cKit. (**G**) Double staining shows that cultured cells coexpress the cardiac stem cell markers CD31 and CD105. (**H**) After cardiac differentiation with biochemical cues, 94% of cells express the cardiomyocyte protein α-actinin. (**I**) After differentiation with biochemical cues, the gap junction protein Connexin43 is expressed at the surface of α-actinin^+^ cells, indicated by arrowheads.

To localize putative adult cardiac stem cell populations within their endogenous niche, human heart auricles (LAA = left atrial appendage) (Figure 1A) were obtained during routine heart surgery. Within this heart auricle tissue, a typical morphology of three main layers was observable: the myocardium is cardiac muscle tissue, mainly cardiomyocytes that are surrounded by the epicardium on the outer surface and the endocardium on the inner surface of the heart (Figure 1B). Immunohistochemistry revealed the presence of cells positive for the neural-crest markers S100B and Nestin in the adult myocardium and not in the endocardium or epicardium (Figure 1C).

### 3.2. Successfully iIsolated Putative Human Cardiac Stem Cells from Heart Auricle Tissue Show High Clonal Efficiency and Capability for Cardiosphere Formation

To analyze the stem cell-marker expressing cells found in the adult human myocardium in more detail, we modified an established protocol for the isolation of human cardiomyocyte progenitor cells [12]. Explant culture resulted in the isolation of cell migrating out of tissue pieces that spontaneously formed cardiospheres. To investigate their stemness characteristics, the clonal growth of putative hCSCs was analyzed. Here, putative hCSCs revealed a clonal efficiency of 22.7%. Importantly, clonally grown cells maintained their ability to form cardiospheres under suspension culture conditions (Figure 1D).

### 3.3. Isolated Cells Express Known Marker Proteins of Cardiac Stem and Progenitor Cells

Characterizing putative hCSCs in more detail, we further observed a high amount of 87% to 95.6% of Sca1+ cells isolated from two distinct hCSC donors but no expression of c-kit using flow cytometry (Figure 1E–G). Multiple staining followed by flow cytometric measurement showed 92.56% of cells being double positive for CD105 and CD31 (Figure 1G), which has also been shown by Smits and colleagues [12].

### 3.4. HCSCs Are Able to Give Rise to Cardiomyocytes In Vitro

To investigate the cardiogenic differentiation potential of the hCSC population, we exposed the cells to TGFβ and ascorbic acid, as published by Smits and colleagues [12]. A high proportion(94%)of α-actinin^+^ cells were detected by immunocytochemistry, indicating a successful cardiac differentiation, whereas no α-actinin was observable in undifferentiated control cells (Figure 1H). Additionally, these cells showed the expression of the gap junction protein Connexin 43, visible as punctual structures (Figure 1I).

### 3.5. Blood Plasma and Blood Serum Strongly Enhance Proliferation-Inducing Effects of Blood Plasma on Adult Human Cardiac Stem Cells

**Figure 2:**
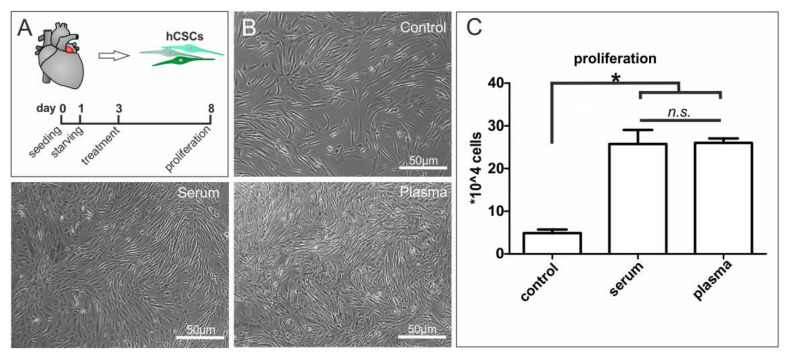
Application of human blood serum and human blood plasma on adult human cardiac stem cells led to increased cell proliferation. (**A**) Primary cultures of human stem cells from the adult heart auricle where exposed to blood serum, blood plasma or starvation medium. (**B**) Microscopy images of treated cells demonstrated a proliferation inducing effect of blood serum and plasma compared to untreated cells. (**C**) Serum and plasma significantly increased the proliferation of hCSCs in a similar manner. Mann-Whitney two-tailed, * *p* < 0.05 was considered significant, not significant (n.s.) *p* > 0.05.

To investigate potential proliferation-inducing effects of blood plasma on hCSCs, we exposed the cells to heparin-treated human blood plasma or human blood serum, which was applied to decrease the number of putative active plasma components (Figure 2A). Control cells cultured in starvation medium showed enlarged and flattened cell morphology, as would be expected of bona fide senescent cells. (Figure 2B, control). In contrast, exposure of hCSCs to human blood plasma or serum resulted in a smaller cell morphology (Figure 2B) next to strongly and significantly increased proliferation (Figure 2C). Notably, no difference was detectable between plasma and serum in the enhancing effects on the proliferation of hCSCs (Figure 2C).

### 3.6. Age and Sex of Blood Serum Donors Do Not Affect Beneficial Effects on Proliferation and Metabolism of hCSCs

**Figure 3:**
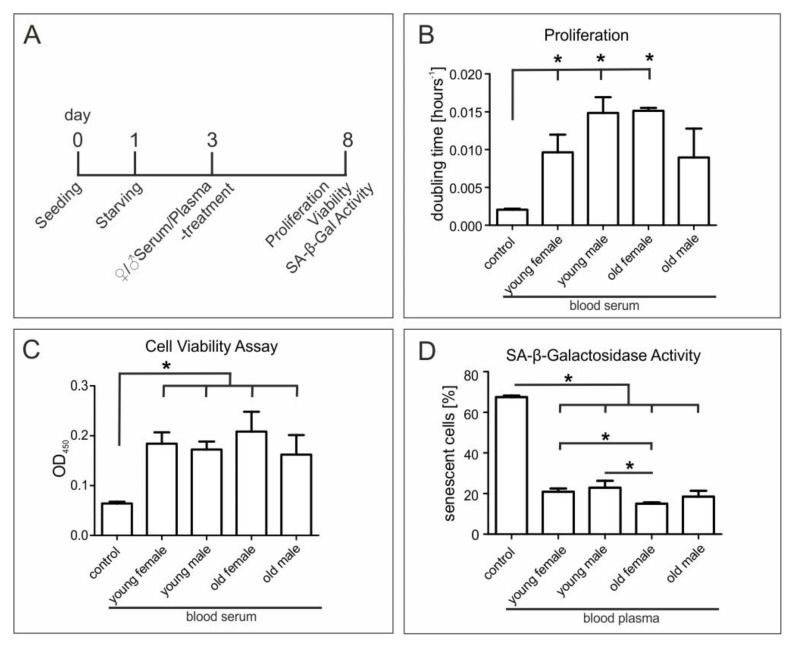
Application of different serum and plasma samples on adult human cardiac stem cells. (**A**) hCSCs were exposed to blood serum and blood plasma from old (>60 years) and young (<20 years) male and female donors or starvation medium. (**B**) Treatment with sera from young female, young male and old female donors significantly increased the proliferation of hCSCs. (**C**) Orangu cell viability assay to measure the metabolism of hCSCs showed increased metabolism after serum treatment but no sex or age dependency. (**D**) SA-β-Galactosidase assay showed a decrease of senescent cells after plasma treatment compared to untreated cells. Mann-Whitney two-tailed, * *p* < 0.05 was considered significant.

Since several studies have suggested an age-dependent effect of blood plasma on stem cell behavior in the murine system [5,33], we applied serum from young (18–20 years) and old (>60 years) female and male donors to hCSCs (Figure 3A). We again observed a strongly increased proliferation of hCSCs treated with human blood serum independent to serum donor age or sex (Figure 3B). Exposure of blood serum from young and old female and male donors further resulted in significantly increased metabolism of hCSCs compared to control, but only modest variations between the serum-treated samples (Figure 3C).

### 3.7. Exposure of hCSCs to Blood Serum from Young Female or Male Donors Results in Significantly Enhanced Protection against Senescence Compared to Serum from Old Female Individuals

We next assessed the ability of blood serum from donors of different ages and sexes to protect hCSCs from starvation-mediated senescence by applying a senescence associated β-galactosidase (SA-β-Gal) activity assay. In comparison to control cells undergoing starvation, blood serum from young female or male donors (18–20 years) and old female or male donors (>60 years) led to significantly and strongly decreased senescence of hCSCs (Figure 3D and Figure S1B). Notably, we observed a significantly enhanced protection against senescence in hCSCs exposed to serum from young female or male individuals compared to serum from old female donors (Figure 3D), suggesting a moderate yet significant age-dependent difference in blood-serum-mediated protection against senescence.

### 3.8. Young Blood Serum Enhances Differential Global Gene Expression of hCSCs

**Figure 4:**
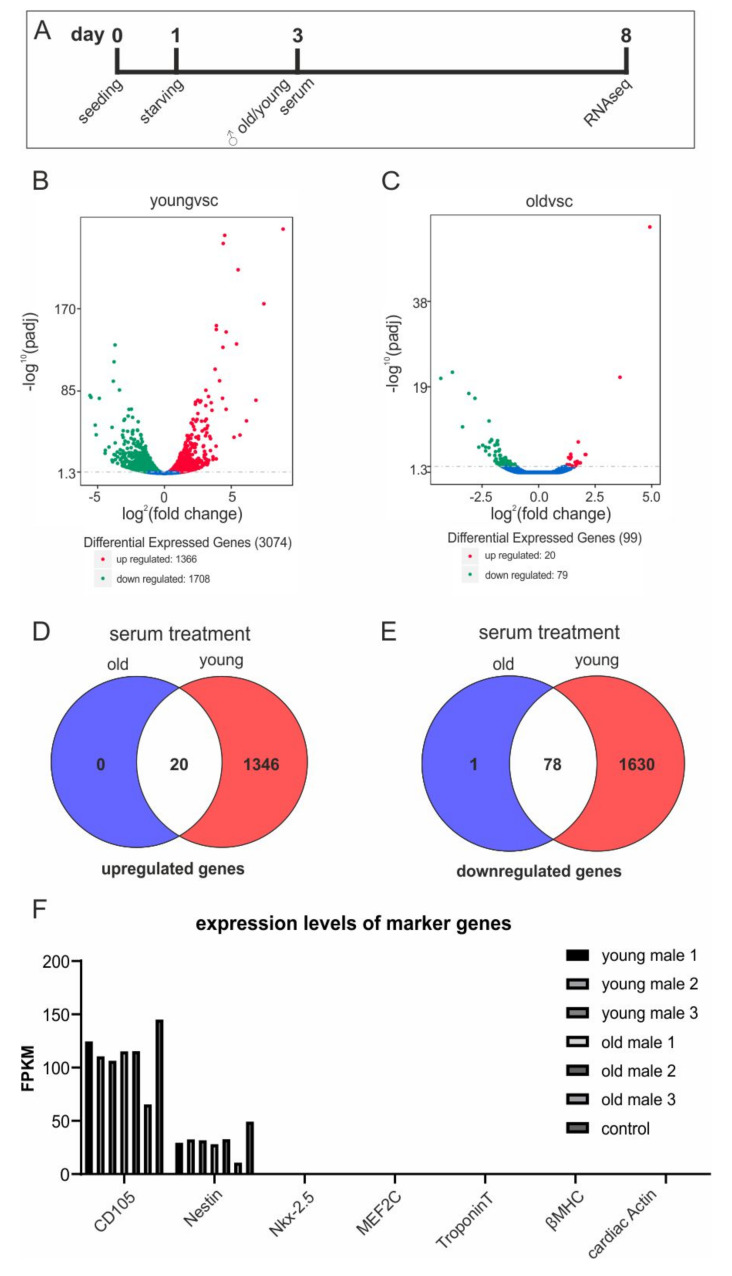
RNAseq showed increased differential gene expression in an age-dependent manner. (**A**) hCSCs where exposed to blood serum and blood plasma from old (>60 years) and young (<20 years) male donors or starvation medium followed by RNAseq. (**B**) Volcano plot of differential expressed genes in hCSCs treated with young serum vs. control. (**C**) Volcano plot of differential expressed genes in hCSCs treated with old serum vs. control. Red: upregulated; green: downregulated genes. A detailed list is provided in Supplementary Table S1. (**D**) Venn diagram of upregulated genes in the old and young treatment groups. (**E**) Venn diagram of downregulated genes in the old and young treatment groups. (**F**) Gene expression levels (in fragments per kilobase million, FPKM) of selected marker genes for cardiac stem cells (CD105, Nestin) and genes that are upregulated during cardiac differentiation (Nkx-2.5, MEF2C, TroponinT, βMHC, cardiac actin). Expressions of CD105 and Nestin are not affected by serum treatment, and cardiac differentiation genes were not expressed either in the control or in the serum-treated samples.

With regard to beneficial effects of blood serum on proliferation of hCSCs and the age-dependent differences observed in protection of hCSCs against senescence, we investigated the effects of blood serum from old and young male donors on global gene expression of hCSCs using RNAseq (Figure 4A). Here, we focused on the examination of potential age-dependent effects of human blood serum on the transcriptome level, since potential differences in the effects of blood serum related to the sex of the donor have not been reported so far. However, the literature frequently describes a rejuvenation phenomenon in the murine system when applying young blood/serum to older individuals.

Differential gene expression analysis between untreated and serum-treated hCSCs revealed a remarkably increased differential gene expression after application of young serum compared to control (Figure 4B) in comparison to old serum compared to control (Figure 4C). In particular, hCSCs treated with young serum showed upregulation of 1366 genes and downregulation of 1708 genes (Figure 4B), while hCSCs treated with serum from old donors differentially upregulated only 20 genes and downregulated 79 genes (Figure 4C). Both treatment groups showed an overlap of 20 upregulated genes (Figure 4D) and 78 downregulated genes (Figure 4E). Importantly, the gene expression levels of the stem cell marker Nestin and the cardiac stem cell marker CD105 remained unaffected after serum treatment. Moreover, genes that were commonly upregulated during cardiac differentiation were not expressed in serum-treated cells (Figure 4F), indicating that hCSCs keep their stem cell-like identity and do not differentiate upon serum treatment. Interestingly, treatment with young serum resulted in a significant reduction of MCP1 expression with a -1.3 fold change compared to untreated cells, whereas eotaxin and GDF11 were not differentially expressed (Supplementary Table S1). Further, IL24 could be found among the most significantly enriched transcripts after treatment with young serum with a log2fold change of +8.8 (Supplementary Table S1). The cytokines GDF11, MCP-1 and eotaxin were frequently discussed in rejuvenation experiments with old mice [34,35,36,37,38,39]. We therefore assessed the respective protein concentrations in old and young plasma samples via ELISA assays (Figure S1C). Neither increases in chemokine and cytokine levels of GDF11, MCP-1 and eotaxin nor increases in proliferation and senescence could be detected when comparing old and young plasma samples.

### 3.9. Global Gene Expression Profiling Indicates Age-Dependent Clusters of Blood-Serum-Treated Adult Stem Cells

**Figure 5:**
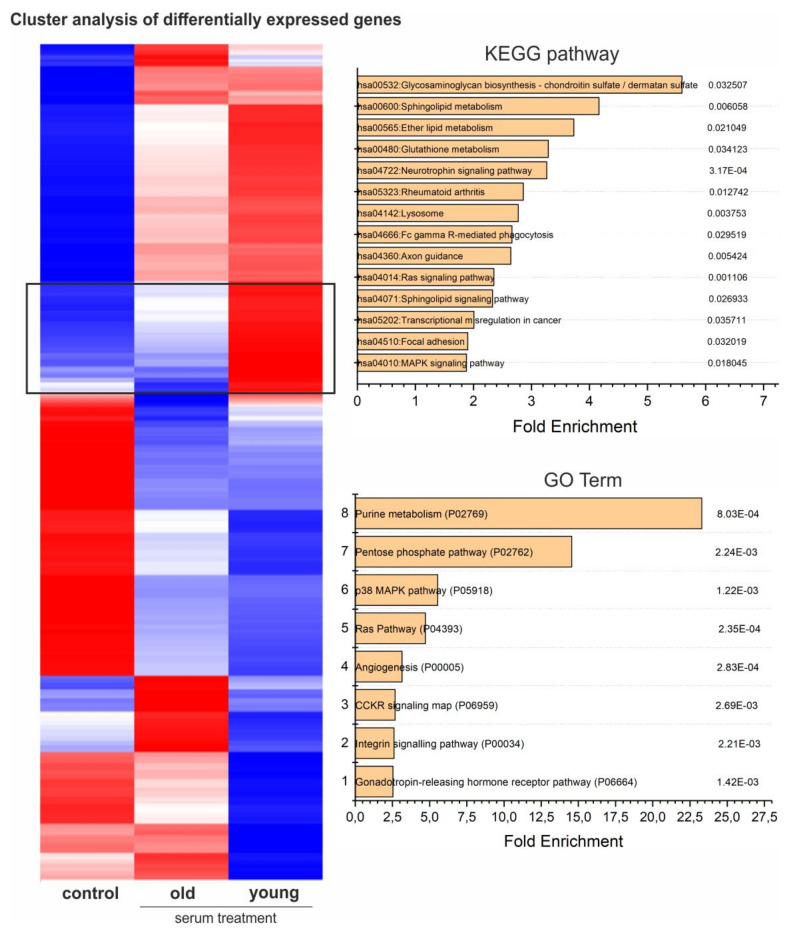
Heatmap of differentially expressed genes with KEGG-pathway analysis and GO-term enrichment of genes highly upregulated in hCSCs treated with young serum and downregulated in hCSCs treated with old serum and in the control (cluster marked with black box).

Using hierarchical clustering of gene expression levels, we generated a heatmap separating the groups of untreated hCSCs and hCSCs treated with either young or old blood serum into distinct clusters. Even though the Pearson correlation analysis showed only marginal differences between the treatment groups (Figure S2), cluster analysis of differentially expressed genes resulted in clear differences along the whole transcriptome (Figure 5, Figures S3 and S4).

### 3.10. Gene Ontology (GO) Term Analysis Reveals Downregulation of Attachment-Associated Genes and Upregulation of Proliferation-Associated GO Terms, Including p38-MAPK

**Figure 6:**
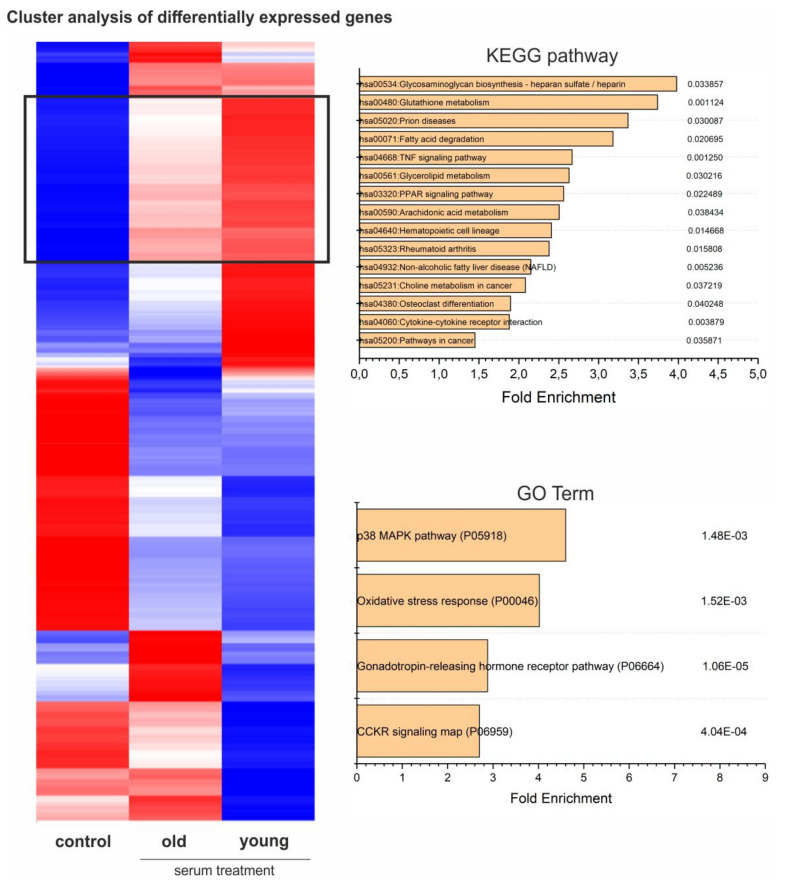
Heatmap of differentially expressed genes with KEGG-pathway analysis and GO-term enrichment of genes highly upregulated in hCSCs treated with young serum, slightly upregulated in hCSCs treated with old serum and downregulated in the control (cluster marked with black box).

Analysis of GO Term enrichment showed beneficial effects on cell cycle and proliferation which was underlined by enrichment of the p38 MAPK pathway (P05918) as the most enriched GO-Term in samples treated with either young or old blood serum (Figure 6). In addition, the term oxidative stress response (P00046) followed as the second most enriched. Interestingly, the application of a KEGG-pathway analysis within this cluster also demonstrated amongst others, the upregulation of the KEGG-pathway glutathione metabolism (hsa00480), further highlighting a possible antioxidative effect of blood serum on hCSCs. In addition, analysis of the genes upregulated only in cells treated with young serum showed GO terms associated with DNA and protein-synthesis-like purine metabolism (P02769) or the pentose phosphate pathway (P02762), leading to enhanced proliferation (Figure 5). The p38 MAPK pathway (P05918) was also in this cluster among the significantly enriched GO terms with a 5.5-fold enrichment and in the KEGG pathways (hsa04010) (Figure 6). Interestingly, the application of GO terms and KEGG-pathway enrichment on a cluster of genes downregulated in cells treated with young serum but upregulated in cells treated with old serum led to the significant enrichment of the GO terms integrin signaling pathway (P00034) and cadherin signaling pathway (P00012) and the KEGG-pathways ECM-receptor interaction (hsa04512), adherens junction (hsa04520), focal adhesion (hsa04510) and tight junction (hsa04530), possibly indicating degenerated exchange with the extracellular matrix (ECM) or neighboring cells or altered intercellular communication—one of the nine hallmarks of aging that were defined in 2013 [4] (Figure S4). Moreover, within this cluster we found the KEGG-pathways arrythmogenic right ventricular cardiomyopathy (hsa05412), hypertrophic cardiomyopathy (hsa05410) and dilated cardiomyopathy (hsa05414), which may indicate an age-dependent protective effect of young blood serum on hCSCs (Figure S4). In summary, GO enrichment analysis revealed the upregulation of various pathways, with p38-MAPK pathway being the most enriched GO term in genes that are highly upregulated after treatment with young serum and not regulated in the old serum group (see Figure 6). Further, p38-MAPK is also in third position (after general metabolism-associated GO terms) of the most enriched GO terms in genes that are highly upregulated after treatment with young serum and downregulated after treatment with old serum (Figure 5).

### 3.11. Beneficial Effects of Blood Plasma on Proliferation and Protection of hCSCs Against Senescence Are Partially Mediated by p38 MAPK-Signaling

**Figure 7:**
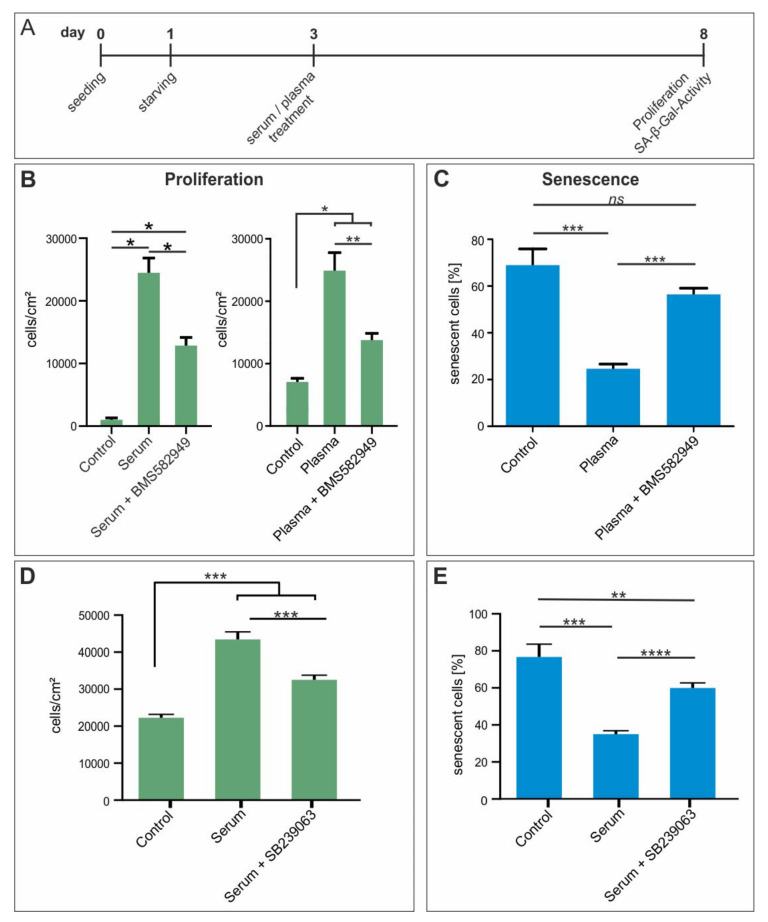
In vitro inhibition of p38-MAPK reversed the beneficial effect of blood plasma on proliferation and survival of hCSCs. (**A**) hCSCs where exposed to blood plasma and serum and p38 inhibitors BMS-582949 and SB239063. (**B**) Inhibition of p38 with BMS582949 led to significantly decreased proliferation compared to plasma and serum treatment alone. (**C**) Inhibition of p38 with BMS582949 led to significantly elevated SA-β-Gal activity compared to plasma treatment alone. (**D**) Inhibition of p38 with SB239063 led to significantly decreased proliferation compared to serum treatment alone. (**E**) Inhibition of p38 with SB239063 led to significantly elevated SA- β-Gal activity compared to serum treatment alone. Mann-Whitney two-tailed, * *p* < 0.05; ** *p* < 0.005; *** *p* < 0.0005; **** *p* < 0.0001 was considered significant, not significant (n.s.) *p* > 0.05.

With regards to the up-regulation of genes associated to p38 MAPK pathway in hCSCs exposed to young serum, we were encouraged to assess its functional role in the before observed effects (Figure 2 and Figure 3). Therefore, we applied two inhibitors of p38 MAPK (BMS-582949 and SB239063) in senescence- and proliferation assays (Figure 7A). Notably, simultaneous exposure of hCSCs to blood plasma and the p38 MAPK inhibitors BMS-582949 and SB239063 led to a strongly decreased proliferation compared to blood plasma or serum-treated hCSCs (Figure 7B,D). Accordingly, we observed a strongly elevated increase in senescence of blood plasma or serum-treated hCSCs after application of the p38 MAPK inhibitors in comparison to hCSCs solely exposed to blood plasma or serum (Figure 7C,E). These results are in line with the upregulation of p38 associated KEGG-pathways and GO terms in hCSCs treated with serum from young male donors (Figure 5 and Figure 6) and emphasize the regulatory role of p38 MAPK in terms of blood-serum-mediated proliferation and protection against senescence.

## 4. Discussion

Ageing is characterized by a decline of homeostatic and regenerative capacities, at least partly caused by the exhaustion of endogenous stem cell functions [4], the accumulation of intracellular ROS and cells undergoing senescence in various tissues and organs. Prominent studies in the murine system suggest that circulating factors play a critical role within this process [5,6,7]. For instance, experiments with heterochronic parabiosis in mice resulted in elevated proliferation of muscle satellite cells, liver, skin, neuronal stem cells and pancreatic β-cells [5,33,40,41]. More rejuvenation strategies targeting the cardiovascular system of aging mice have been recently reviewed by Cesselli et al. [42]. Besides, other studies provided indirect evidence by transplanting islet-cells from young or old mice into hyperglycemic recipients resulting in similar replication rates of both old and young donor cells [43]. However, a rejuvenation of aged human cells or organs by the application of a young systemic milieu could not be shown so far [8]. In the present study, we compare the effects of human blood plasma and serum of young and old donors on adult cardiac stem cells from the human heart auricle. Although the adult heart was long ago considered a terminally differentiated organ with low regenerative potential, rare populations of mouse and human cardiac stem cells (CSCs) were found that may contribute to endogenous repair mechanisms [10,11,12]. Although identification and isolation of adult CSCs often utilize the expression of the cell-surface marker c-kit [10,15,16], expression was also reported in non-cardiac cells [44,45,46] and its relevance as a cardiac stem cell marker is controversially discussed. For instance, Sultana and co-workers showed that c-kit^+^ cells in the mouse heart are endothelial cells and not cardiac stem cells [47], and c-kit-negative stem cell populations have already been described in the human heart [11]. Interestingly, Tomita et al. reported that Nestin^+^ NCSCs in the mouse heart give rise to cardiomyocytes in vivo [48]. Accordingly, we show here that the myocardium of the adult human heart auricle contains a population of Nestin^+^/S100B^+^ cardiac stem cells positive for Sca1 and CD105 but lacking c-kit-expression. Next to c-kit, the surface antigens Sca1 and CD105 are commonly used markers for human cardiac stem and progenitor cells [12]. In contrast to most studies showing co-expression of Sca1, c-kit and CD105 in human CSCs [12], we observed the presence of Sca1 and CD105 in human cardiac stem cells despite the lack of c-kit. Isolated hCSCs were able to grow clonally while maintaining their capability of sphere formation and differentiated efficiently into α-actinin^+^ cardiomyocyte-like cells. Although the application of human blood serum, blood plasma or platelet-rich plasma on stem cell cultures has already been shown to effectively promote cell proliferation [21,22,49,50,51,52,53], the cardiovascular system and especially cardiac stem cells have not been investigated so far. Within this study we likewise could demonstrate an overall enhancing effect of human blood serum on adult human cardiac stem cells, while no age dependency could be detected in terms of proliferation and metabolic activity. In line with these results, other studies focusing on data analysis of red blood cell transfusions in terms of donor age and sex and the survival rates of the transfusion-recipients have not shown an age or sex-related effect [54]. Importantly, we could not detect significant changes in the gene expression levels of CD105 and Nestin upon serum-treatment, nor the expression of cardiac differentiation genes in any of the samples. These results demonstrate that hCSCs keep their stem cell-like features and do not differentiate spontaneously during exposure to blood serum. Accordingly, other adult stem cell populations were also reported to keep their characteristic gene expression and stemness characteristics when cultivated in human blood plasma [22,55].

To investigate the underlying molecular mechanisms driven by the application of blood serum, we performed RNAseq and compared the global transcriptome of hCSCs treated with either old (donor age >60 years) or young (donor age <20 years) male blood serum. This state-of-the-art technique allowed us to analyze the highly complex regulatory networks that drive proliferation of human cardiac stem cells. Here, we focused on the examination of potential age-dependent effects, since sex-specific differences were not detectable on proliferation or senescence of serum-treated hCSCs. In line with these findings, potential differences in the effects of blood serum related to the sex of the donor have not been reported so far. However, the literature frequently describes a rejuvenation phenomenon in the murine system when applying young blood/serum to old individuals [5,6,7]. Our present observations show that the application of young blood serum leads to an increased number of up or downregulated genes compared to the treatment with old blood serum. To the best of our knowledge, comparable transcriptomic data of human cells after exposure to serum or plasma do not exist. However, a general decline in global gene expression of aging tissues has been reported in several studies [56,57,58,59,60], which is line with our present findings. In detail, the pattern of differentially expressed genes seems to be dependent on tissue type. For instance, Lipinski and colleagues showed that genes associated with autophagy are downregulated in the human brain during aging [59]. Other groups showed differential gene expression of migration and proliferation-associated genes in male skin samples with advanced age [60]. We likewise could show increased differential gene expression in hCSCs upon the treatment with young blood serum compared to the application of old blood serum. However, this seems to have no further beneficial influence on the effects on cell proliferation and viability compared to the application of old serum. We therefore suggest that both old and young serum trigger activation of pathways leading to enhanced proliferation and protection against senescence. Here, our analysis identified p38MAPK as a crucial pathway for regulating proliferation and senescence in a blood-serum-dependent manner, as discussed in detail below. In accordance to our observations, human blood serum, blood plasma or platelet-rich plasma were reported to be beneficial for proliferation of stem cells, despite the age of the plasma donors [21,22,56,57,58,59,60]. The highly elevated global gene expression levels after treatment of hCSCs with young blood serum compared to old serum further suggest that the application of young serum may result in cellular effects other than proliferation or protection against senescence. Considering this discrepancy in our observation, a more detailed transcriptomic analysis was necessary to understand age-related blood borne effects on cultured cells. A detailed analysis of upregulated genes after serum treatment showed significant overrepresentation of cell cycle and proliferation-enhancing GO terms, which is in line with the observed beneficial effects of blood serum on proliferation of hCSCs. Moreover, we could observe the upregulation of integrin signaling and cadherin signaling, which is also connected to proliferation in several instances [61]. In addition, a range of studies also suggest circulating pro-ageing factors with the pro-inflammatory chemokine MCP1 as one of the most popular candidates. Level of MCP1 increases with age in mice and humans and is even discussed as a biomarker for cardiac aging [34,36]. Accordingly, we could show via RNAseq that MCP1 was significantly downregulated in cells treated with young blood serum. Further, Ghosh and coworkers recently showed that levels of MCP1 in white adipose tissue in old mice were decreased upon heterochronic parabiosis with young mice and even in cell culture after conditioning with young serum [62]. In contrast to this, we detected no significant differences in the protein concentration of MCP1 between old and young plasma samples. Next to MCP1, other factors like GDF11 or eotaxin (CCL11) are also discussed as age-dependent blood borne cytokines [35,37,38,39]. These chemokines and cytokines were measured in moderate yet significantly higher abundances in young plasma samples compared to old plasma samples. However, our data did not show a differential expression of the corresponding genes in serum-treated hCSCs. These differences between protein concentrations in the plasma samples and gene expression levels in the treated cells may be partially explained by the only modest changes of protein contents that are not sufficient to trigger differential gene expression.

Notably, analysis of the global gene expression profile of hCSCs treated with young or old blood serum showed the upregulation and enrichment of genes in the p38-MAPK-associated GO term P05918 and KEGG-pathway hsa04010. In general, p38-MAPK signaling is understood as an inhibitor of proliferation [63] but it has also been described to enhance proliferation in a range of cell types [64,65], suggesting that the role of p38-MAPK in proliferation and senescence is strongly cell-specific. For example, in male human skin samples, MAPK signaling is upregulated along with cell proliferation in aged (>70 years) patients [60]. Further, in human breast cancer cells, p38 MAPK upregulation is associated with increased proliferation and can be inhibited by the use of the p38 inhibitor SB203580 or p38α-siRNA [64,66]. In line with these observations, inhibition of p38 significantly reversed the beneficial effects of blood serum in terms of proliferation and protection from senescence in the present study. Interestingly, we also detected the cytokine IL24 to be highly upregulated in hCSCs after treatment with young blood serum. IL24 was reported to induce p38 MAPK activation [67], suggesting a role in the upstream regulation of p38 in hCSCs in a blood-serum-dependent manner. However, other relevant signaling pathways may interact with p38 MAPK in controlling blood-serum-mediated proliferation of hCSCs. For instance, other MAPKs, such as ERK, were described to co-regulate proliferation of stem cells together with p38 MAPK in response to stimuli such as hypoxia or proliferation-inducing drugs [68,69]. In particular, treatment of periodontal ligament stem cells with inhibitors against p38 MAPK or ERK resulted in reduced hypoxia-mediated proliferation [69]. Likewise, signaling via p38 MAPK and NF-κB was described to co-regulate proliferation of hepatic stem cells [70] and also has an essential role in the regulation of myocardial adaption to ischemia [71]. Accordingly, our present data reveal a relevant role of p38 MAPK in regulating proliferation of adult human cardiac stem cells. Regarding the reversion of the senescent phenotype after blood plasma treatment, Liu and colleagues likewise showed that young blood plasma reverses age-dependent senescence in hepatic tissues of rodents. This effect was not reported in application of old plasma [72]. Other studies also describe similar effects with young blood on various organs in heterochronic parabiotic mice [5,35,62,73,74]. However, our results show a more general protective effect of human blood plasma against senescence which is independent of age and sex. These contrasting results also demonstrate the difficulties in the transition from the murine to the human system and the highly complex regulation of the ageing process. Here, next to the investigation of age-dependent effects upon usage of human blood plasma or serum, the cell types or tissues that are addressed should also be taken into account carefully—possibly with the examination of p38-MAPK as a crucial regulator.

In summary, we demonstrate within this study the beneficial effects of blood serum on the proliferation and metabolism of adult human cardiac stem cells, which are accompanied by a decrease of senescent cells. By the application of RNAseq we were able to describe the changes in the global gene expression profiles of serum-treated hCSCs and thus successfully identified the age-dependent enhancement of p38-MAPK signaling as one of the underlying pathways that promote the blood-serum-mediated proliferation.

## Supplementary Materials

The following are available online at https://www.mdpi.com/2073-4409/9/6/1472/s1. Figure S1: (A) Application of different individual serum samples in a proliferation assay. (B) Application of different individual plasma samples in a senescence assay with adult human cardiac stem cells. (C) ELISA-measurements of the plasma content of the chemokines and cytokines eotaxin 1. GDF11 and MCP-1, Figure S2: Pearson Correlation of global gene expression profiles of hCSCs treated with either young or old serum and of the starvation control, Figure S3: Heatmap of differentially expressed genes with KEGG-pathway analysis and GO-term enrichment of genes downregulated in hCSCs treated with young serum and old serum and strongly upregulated in the control, Figure S4: Heatmap of differentially expressed genes with KEGG-pathway analysis and GO-term enrichment of genes strongly downregulated in hCSCs treated with young serum and upregulated in hCSCs treated with old serum, Table S1: List of differentially expressed genes that are shown in Figure 3B,C.

## References

[1] Laflamme M.A., Murry C.E. (2011). Heart regeneration. Nature.

[2] Aguilar-Sanchez C., Michael M., Pennings S. (2018). Cardiac Stem Cells in the Postnatal Heart: Lessons from Development. Stem Cells Int..

[3] Franceschi C., Garagnani P., Morsiani C., Conte M., Santoro A., Grignolio A., Monti D., Capri M., Salvioli S. (2018). The Continuum of Aging and Age-Related Diseases: Common Mechanisms but Different Rates. Front. Med. (Lausanne).

[4] Lopez-Otin C., Blasco M.A., Partridge L., Serrano M., Kroemer G. (2013). The hallmarks of aging. Cell.

[5] Conboy I.M., Conboy M.J., Wagers A.J., Girma E.R., Weissman I.L., Rando T.A. (2005). Rejuvenation of aged progenitor cells by exposure to a young systemic environment. Nature.

[6] Villeda S.A., Plambeck K.E., Middeldorp J., Castellano J.M., Mosher K.I., Luo J., Smith L.K., Bieri G., Lin K., Berdnik D. (2014). Young blood reverses age-related impairments in cognitive function and synaptic plasticity in mice. Nat. Med..

[7] Castellano J.M., Mosher K.I., Abbey R.J., McBride A.A., James M.L., Berdnik D., Shen J.C., Zou B., Xie X.S., Tingle M. (2017). Human umbilical cord plasma proteins revitalize hippocampal function in aged mice. Nature.

[8] Hofmann B. (2018). Young Blood Rejuvenates Old Bodies: A Call for Reflection when Moving from Mice to Men. Transfus. Med. Hemother..

[9] Sha S.J., Deutsch G.K., Tian L., Richardson K., Coburn M., Gaudioso J.L., Marcal T., Solomon E., Boumis A., Bet A. (2019). Safety, Tolerability, and Feasibility of Young Plasma Infusion in the Plasma for Alzheimer Symptom Amelioration Study: A Randomized Clinical Trial. JAMA Neurol..

[10] Beltrami A.P., Barlucchi L., Torella D., Baker M., Limana F., Chimenti S., Kasahara H., Rota M., Musso E., Urbanek K. (2003). Adult cardiac stem cells are multipotent and support myocardial regeneration. Cell.

[11] Koninckx R., Daniels A., Windmolders S., Mees U., Macianskiene R., Mubagwa K., Steels P., Jamaer L., Dubois J., Robic B. (2013). The cardiac atrial appendage stem cell: A new and promising candidate for myocardial repair. Cardiovasc. Res..

[12] Smits A.M., van Vliet P., Metz C.H., Korfage T., Sluijter J.P., Doevendans P.A., Goumans M.J. (2009). Human cardiomyocyte progenitor cells differentiate into functional mature cardiomyocytes: An in vitro model for studying human cardiac physiology and pathophysiology. Nat. Protoc..

[13] Zaruba M.M., Soonpaa M., Reuter S., Field L.J. (2010). Cardiomyogenic potential of C-kit(+)-expressing cells derived from neonatal and adult mouse hearts. Circulation.

[14] Tallini Y.N., Greene K.S., Craven M., Spealman A., Breitbach M., Smith J., Fisher P.J., Steffey M., Hesse M., Doran R.M. (2009). c-kit expression identifies cardiovascular precursors in the neonatal heart. Proc. Natl. Acad. Sci. USA.

[15] Bearzi C., Rota M., Hosoda T., Tillmanns J., Nascimbene A., De Angelis A., Yasuzawa-Amano S., Trofimova I., Siggins R.W., Lecapitaine N. (2007). Human cardiac stem cells. Proc. Natl. Acad. Sci. USA.

[16] Urbanek K., Cesselli D., Rota M., Nascimbene A., De Angelis A., Hosoda T., Bearzi C., Boni A., Bolli R., Kajstura J. (2006). Stem cell niches in the adult mouse heart. Proc. Natl. Acad. Sci. USA.

[17] Moretti A., Caron L., Nakano A., Lam J.T., Bernshausen A., Chen Y., Qyang Y., Bu L., Sasaki M., Martin-Puig S. (2006). Multipotent embryonic isl1+ progenitor cells lead to cardiac, smooth muscle, and endothelial cell diversification. Cell.

[18] Oh H., Bradfute S.B., Gallardo T.D., Nakamura T., Gaussin V., Mishina Y., Pocius J., Michael L.H., Behringer R.R., Garry D.J. (2003). Cardiac progenitor cells from adult myocardium: Homing, differentiation, and fusion after infarction. Proc. Natl. Acad. Sci. USA.

[19] Eschenhagen T., Bolli R., Braun T., Field L.J., Fleischmann B.K., Frisen J., Giacca M., Hare J.M., Houser S., Lee R.T. (2017). Cardiomyocyte Regeneration: A Consensus Statement. Circulation.

[20] Vogel J.P., Szalay K., Geiger F., Kramer M., Richter W., Kasten P. (2006). Platelet-rich plasma improves expansion of human mesenchymal stem cells and retains differentiation capacity and in vivo bone formation in calcium phosphate ceramics. Platelets.

[21] Walenda T., Bokermann G., Jost E., Galm O., Schellenberg A., Koch C.M., Piroth D.M., Drescher W., Brummendorf T.H., Wagner W. (2011). Serum after autologous transplantation stimulates proliferation and expansion of human hematopoietic progenitor cells. PLoS ONE.

[22] Greiner J.F., Hauser S., Widera D., Muller J., Qunneis F., Zander C., Martin I., Mallah J., Schuetzmann D., Prante C. (2011). Efficient animal-serum free 3D cultivation method for adult human neural crest-derived stem cell therapeutics. Eur. Cell Mater..

[23] Yamaguchi M., Hirayama F., Wakamoto S., Fujihara M., Murahashi H., Sato N., Ikebuchi K., Sawada K., Koike T., Kuwabara M. (2002). Bone marrow stromal cells prepared using AB serum and bFGF for hematopoietic stem cells expansion. Transfusion.

[24] Hauser S., Widera D., Qunneis F., Muller J., Zander C., Greiner J., Strauss C., Luningschror P., Heimann P., Schwarze H. (2012). Isolation of novel multipotent neural crest-derived stem cells from adult human inferior turbinate. Stem Cells Dev..

[25] Schneider C.A., Rasband W.S., Eliceiri K.W. (2012). NIH Image to ImageJ: 25 years of image analysis. Nat. Methods.

[26] Debacq-Chainiaux F., Erusalimsky J.D., Campisi J., Toussaint O. (2009). Protocols to detect senescence-associated beta-galactosidase (SA-betagal) activity, a biomarker of senescent cells in culture and in vivo. Nat. Protoc..

[27] Dennis G., Sherman B.T., Hosack D.A., Yang J., Gao W., Lane H.C., Lempicki R.A. (2003). DAVID: Database for Annotation, Visualization, and Integrated Discovery. Genome Biol..

[28] Mi H., Muruganujan A., Thomas P.D. (2013). PANTHER in 2013: Modeling the evolution of gene function, and other gene attributes, in the context of phylogenetic trees. Nucleic Acids Res..

[29] Mi H., Muruganujan A., Ebert D., Huang X., Thomas P.D. (2019). PANTHER version 14: More genomes, a new PANTHER GO-slim and improvements in enrichment analysis tools. Nucleic Acids Res..

[30] Mi H., Thomas P. (2009). PANTHER pathway: An ontology-based pathway database coupled with data analysis tools. Methods Mol. Biol..

[31] Wu J., Mao X., Cai T., Luo J., Wei L. (2006). KOBAS server: A web-based platform for automated annotation and pathway identification. Nucleic Acids Res..

[32] Xie C., Mao X., Huang J., Ding Y., Wu J., Dong S., Kong L., Gao G., Li C.Y., Wei L. (2011). KOBAS 2.0: A web server for annotation and identification of enriched pathways and diseases. Nucleic Acids Res..

[33] Villeda S.A., Luo J., Mosher K.I., Zou B., Britschgi M., Bieri G., Stan T.M., Fainberg N., Ding Z., Eggel A. (2011). The ageing systemic milieu negatively regulates neurogenesis and cognitive function. Nature.

[34] Yousefzadeh M.J., Schafer M.J., Noren Hooten N., Atkinson E.J., Evans M.K., Baker D.J., Quarles E.K., Robbins P.D., Ladiges W.C., LeBrasseur N.K. (2018). Circulating levels of monocyte chemoattractant protein-1 as a potential measure of biological age in mice and frailty in humans. Aging Cell.

[35] Loffredo F.S., Steinhauser M.L., Jay S.M., Gannon J., Pancoast J.R., Yalamanchi P., Sinha M., Dall’Osso C., Khong D., Shadrach J.L. (2013). Growth differentiation factor 11 is a circulating factor that reverses age-related cardiac hypertrophy. Cell.

[36] Chiao Y.A., Dai Q., Zhang J., Lin J., Lopez E.F., Ahuja S.S., Chou Y.M., Lindsey M.L., Jin Y.F. (2011). Multi-analyte profiling reveals matrix metalloproteinase-9 and monocyte chemotactic protein-1 as plasma biomarkers of cardiac aging. Circ. Cardiovasc. Genet..

[37] Poggioli T., Vujic A., Yang P., Macias-Trevino C., Uygur A., Loffredo F.S., Pancoast J.R., Cho M., Goldstein J., Tandias R.M. (2016). Circulating Growth Differentiation Factor 11/8 Levels Decline With Age. Circ. Res..

[38] Hoefer J., Luger M., Dal-Pont C., Culig Z., Schennach H., Jochberger S. (2017). The “Aging Factor” Eotaxin-1 (CCL11) Is Detectable in Transfusion Blood Products and Increases with the Donor’s Age. Front. Aging Neurosci..

[39] Egerman M.A., Cadena S.M., Gilbert J.A., Meyer A., Nelson H.N., Swalley S.E., Mallozzi C., Jacobi C., Jennings L.L., Clay I. (2015). GDF11 Increases with Age and Inhibits Skeletal Muscle Regeneration. Cell Metab..

[40] Song G., Nguyen D.T., Pietramaggiori G., Scherer S., Chen B., Zhan Q., Ogawa R., Yannas I.V., Wagers A.J., Orgill D.P. (2010). Use of the parabiotic model in studies of cutaneous wound healing to define the participation of circulating cells. Wound Repair Regen..

[41] Salpeter S.J., Khalaileh A., Weinberg-Corem N., Ziv O., Glaser B., Dor Y. (2013). Systemic regulation of the age-related decline of pancreatic beta-cell replication. Diabetes.

[42] Cesselli D., Aleksova A., Mazzega E., Caragnano A., Beltrami A.P. (2018). Cardiac stem cell aging and heart failure. Pharmacol. Res..

[43] Chen X., Zhang X., Chen F., Larson C.S., Wang L.J., Kaufman D.B. (2009). Comparative study of regenerative potential of beta cells from young and aged donor mice using a novel islet transplantation model. Transplantation.

[44] Morita N., Yamamoto M., Tanizawa T. (2003). Correlation of c-kit expression and cell cycle regulation by transforming growth factor-beta in CD34+ CD38- human bone marrow cells. Eur. J. Haematol..

[45] Rusu M.C., Duta I., Didilescu A.C., Vrapciu A.D., Hostiuc S., Anton E. (2014). Precursor and interstitial Cajal cells in the human embryo liver. Rom. J. Morphol. Embryol..

[46] Ilie C.A., Rusu M.C., Didilescu A.C., Motoc A.G., Mogoanta L. (2015). Embryonic hematopoietic stem cells and interstitial Cajal cells in the hindgut of late stage human embryos: Evidence and hypotheses. Ann Anat.

[47] Sultana N., Zhang L., Yan J., Chen J., Cai W., Razzaque S., Jeong D., Sheng W., Bu L., Xu M. (2015). Resident c-kit(+) cells in the heart are not cardiac stem cells. Nat. Commun..

[48] Tomita Y., Matsumura K., Wakamatsu Y., Matsuzaki Y., Shibuya I., Kawaguchi H., Ieda M., Kanakubo S., Shimazaki T., Ogawa S. (2005). Cardiac neural crest cells contribute to the dormant multipotent stem cell in the mammalian heart. J. Cell Biol..

[49] Pandey S., Hickey D.U., Drum M., Millis D.L., Cekanova M. (2019). Platelet-rich plasma affects the proliferation of canine bone marrow-derived mesenchymal stromal cells in vitro. BMC Vet. Res..

[50] Shen J., Gao Q., Zhang Y., He Y. (2015). Autologous plateletrich plasma promotes proliferation and chondrogenic differentiation of adiposederived stem cells. Mol. Med. Rep..

[51] Shetty P., Bharucha K., Tanavde V. (2007). Human umbilical cord blood serum can replace fetal bovine serum in the culture of mesenchymal stem cells. Cell Biol. Int..

[52] Witzeneder K., Lindenmair A., Gabriel C., Holler K., Theiss D., Redl H., Hennerbichler S. (2013). Human-derived alternatives to fetal bovine serum in cell culture. Transfus. Med. Hemother..

[53] Schürmann M., Brotzmann V., Butow M., Greiner J., Höving A., Kaltschmidt C., Kaltschmidt B., Sudhoff H. (2018). Identification of a Novel High Yielding Source of Multipotent Adult Human Neural Crest-Derived Stem Cells. Stem Cell Rev..

[54] Edgren G., Ullum H., Rostgaard K., Erikstrup C., Sartipy U., Holzmann M.J., Nyren O., Hjalgrim H. (2017). Association of Donor Age and Sex With Survival of Patients Receiving Transfusions. JAMA Intern. Med..

[55] Greiner J.F., Grunwald L.M., Muller J., Sudhoff H., Widera D., Kaltschmidt C., Kaltschmidt B. (2014). Culture bag systems for clinical applications of adult human neural crest-derived stem cells. Stem Cell Res. Ther..

[56] Stegeman R., Weake V.M. (2017). Transcriptional Signatures of Aging. J. Mol. Biol..

[57] Peters M.J., Joehanes R., Pilling L.C., Schurmann C., Conneely K.N., Powell J., Reinmaa E., Sutphin G.L., Zhernakova A., Schramm K. (2015). The transcriptional landscape of age in human peripheral blood. Nat. Commun..

[58] Berchtold N.C., Cribbs D.H., Coleman P.D., Rogers J., Head E., Kim R., Beach T., Miller C., Troncoso J., Trojanowski J.Q. (2008). Gene expression changes in the course of normal brain aging are sexually dimorphic. Proc. Natl. Acad. Sci. USA.

[59] Lipinski M.M., Zheng B., Lu T., Yan Z., Py B.F., Ng A., Xavier R.J., Li C., Yankner B.A., Scherzer C.R. (2010). Genome-wide analysis reveals mechanisms modulating autophagy in normal brain aging and in Alzheimer’s disease. Proc. Natl. Acad. Sci. USA.

[60] Haustead D.J., Stevenson A., Saxena V., Marriage F., Firth M., Silla R., Martin L., Adcroft K.F., Rea S., Day P.J. (2016). Transcriptome analysis of human ageing in male skin shows mid-life period of variability and central role of NF-kappaB. Sci. Rep..

[61] Moreno-Layseca P., Streuli C.H. (2014). Signalling pathways linking integrins with cell cycle progression. Matrix Biol..

[62] Ghosh A.K., O’Brien M., Mau T., Qi N., Yung R. (2019). Adipose Tissue Senescence and Inflammation in Aging is Reversed by the Young Milieu. J. Gerontol. A Biol. Sci. Med. Sci..

[63] Saika S., Okada Y., Miyamoto T., Yamanaka O., Ohnishi Y., Ooshima A., Liu C.Y., Weng D., Kao W.W. (2004). Role of p38 MAP kinase in regulation of cell migration and proliferation in healing corneal epithelium. Invest. Ophthalmol. Vis. Sci..

[64] Chen L., Mayer J.A., Krisko T.I., Speers C.W., Wang T., Hilsenbeck S.G., Brown P.H. (2009). Inhibition of the p38 kinase suppresses the proliferation of human ER-negative breast cancer cells. Cancer Res..

[65] Zarubin T., Han J. (2005). Activation and signaling of the p38 MAP kinase pathway. Cell Res..

[66] Huth H.W., Santos D.M., Gravina H.D., Resende J.M., Goes A.M., de Lima M.E., Ropert C. (2017). Upregulation of p38 pathway accelerates proliferation and migration of MDA-MB-231 breast cancer cells. Oncol. Rep..

[67] Tian H., Zhang D., Gao Z., Li H., Zhang B., Zhang Q., Li L., Cheng Q., Pei D., Zheng J. (2014). MDA-7/IL-24 inhibits Nrf2-mediated antioxidant response through activation of p38 pathway and inhibition of ERK pathway involved in cancer cell apoptosis. Cancer Gene Ther..

[68] Qin S., Zhou W., Liu S., Chen P., Wu H. (2015). Icariin stimulates the proliferation of rat bone mesenchymal stem cells via ERK and p38 MAPK signaling. Int. J. Clin. Exp. Med..

[69] He Y., Jian C.X., Zhang H.Y., Zhou Y., Wu X., Zhang G., Tan Y.H. (2016). Hypoxia enhances periodontal ligament stem cell proliferation via the MAPK signaling pathway. Genet. Mol. Res..

[70] Yao P., Zhan Y., Xu W., Li C., Yue P., Xu C., Hu D., Qu C.K., Yang X. (2004). Hepatocyte growth factor-induced proliferation of hepatic stem-like cells depends on activation of NF-kappaB. J. Hepatol..

[71] Maulik N., Sato M., Price B.D., Das D.K. (1998). An essential role of NFkappaB in tyrosine kinase signaling of p38 MAP kinase regulation of myocardial adaptation to ischemia. FEBS Lett..

[72] Liu A., Guo E., Yang J., Yang Y., Liu S., Jiang X., Hu Q., Dirsch O., Dahmen U., Zhang C. (2018). Young plasma reverses age-dependent alterations in hepatic function through the restoration of autophagy. Aging Cell.

[73] Ruckh J.M., Zhao J.W., Shadrach J.L., van Wijngaarden P., Rao T.N., Wagers A.J., Franklin R.J. (2012). Rejuvenation of regeneration in the aging central nervous system. Cell Stem Cell.

[74] Katsimpardi L., Litterman N.K., Schein P.A., Miller C.M., Loffredo F.S., Wojtkiewicz G.R., Chen J.W., Lee R.T., Wagers A.J., Rubin L.L. (2014). Vascular and neurogenic rejuvenation of the aging mouse brain by young systemic factors. Science.

