# Supplementary figures and images for: Blood Serum Stimulates p38-Mediated Proliferation and Changes in Global Gene Expression of Adult Human Cardiac Stem Cells
Source: Cells. 2020 Jun 16;9(6):1472. doi: 10.3390/cells9061472 (PMC7349155; doi:10.3390/cells9061472)

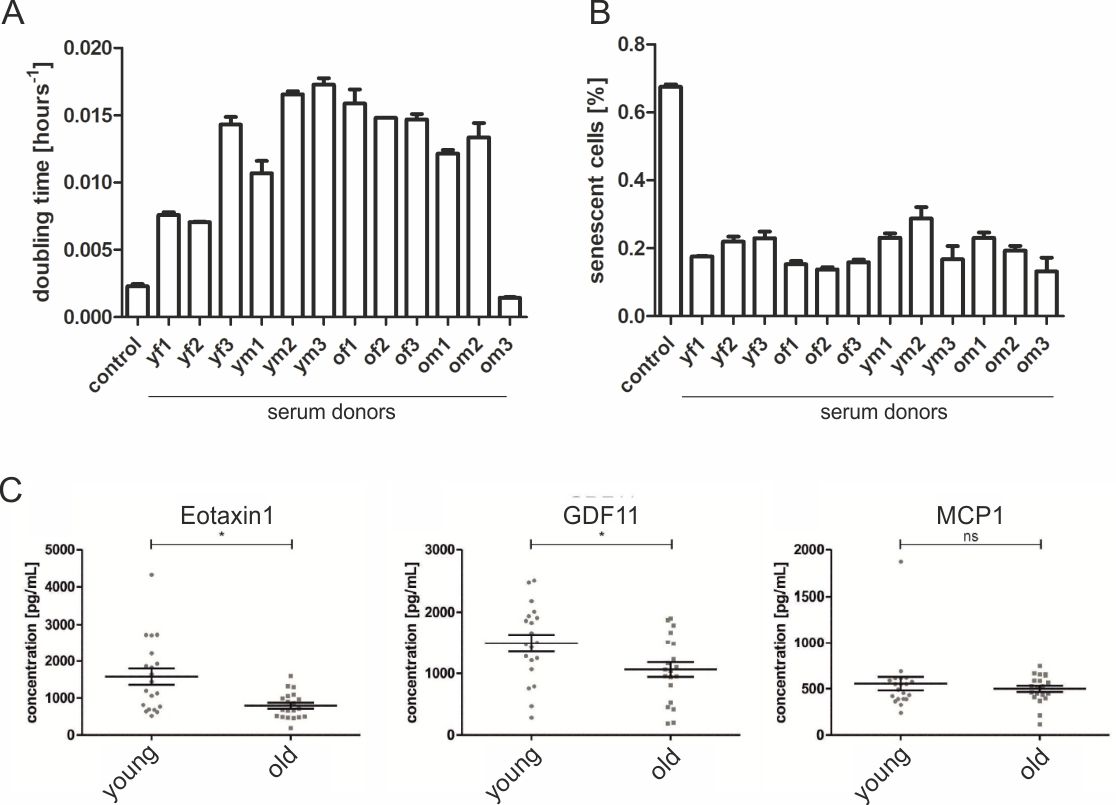

Supplement: Supplementary file 1 [file cells-09-01472-s001.zip › Supplemental material/Figure S1.jpg]

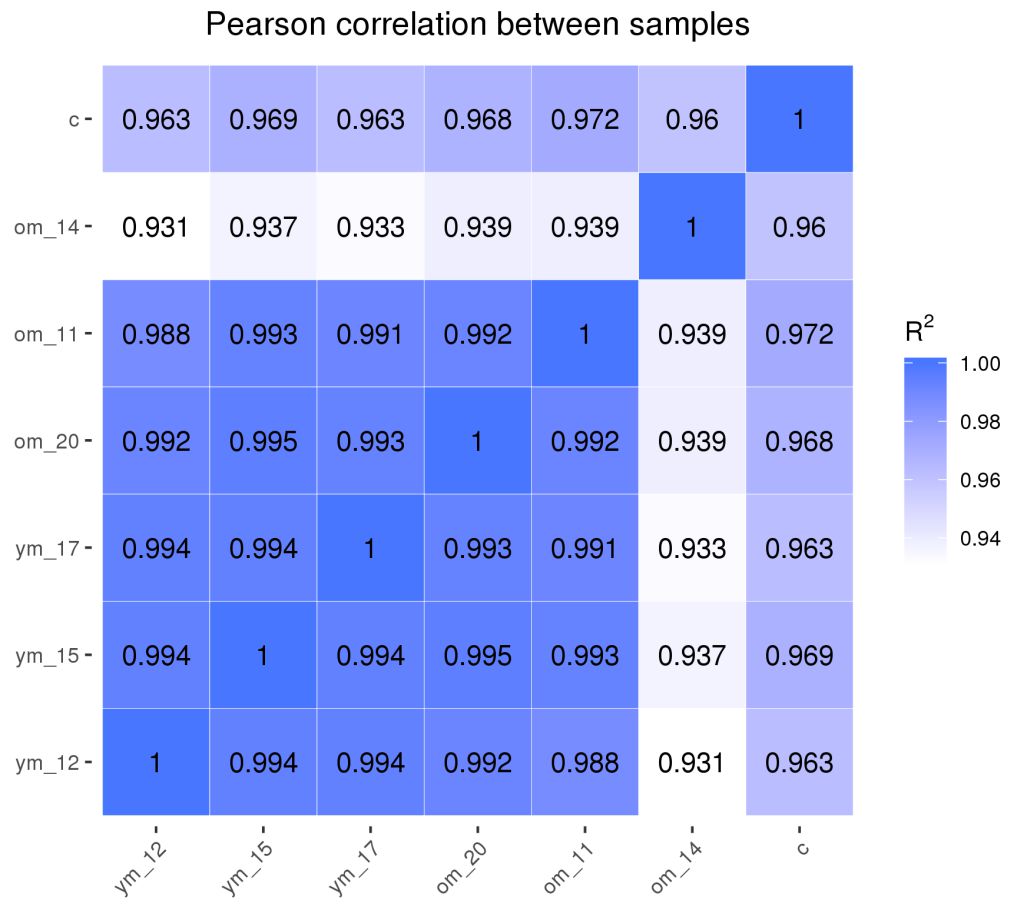

Supplement: Supplementary file 1 [file cells-09-01472-s001.zip › Supplemental material/Figure S2.jpg]

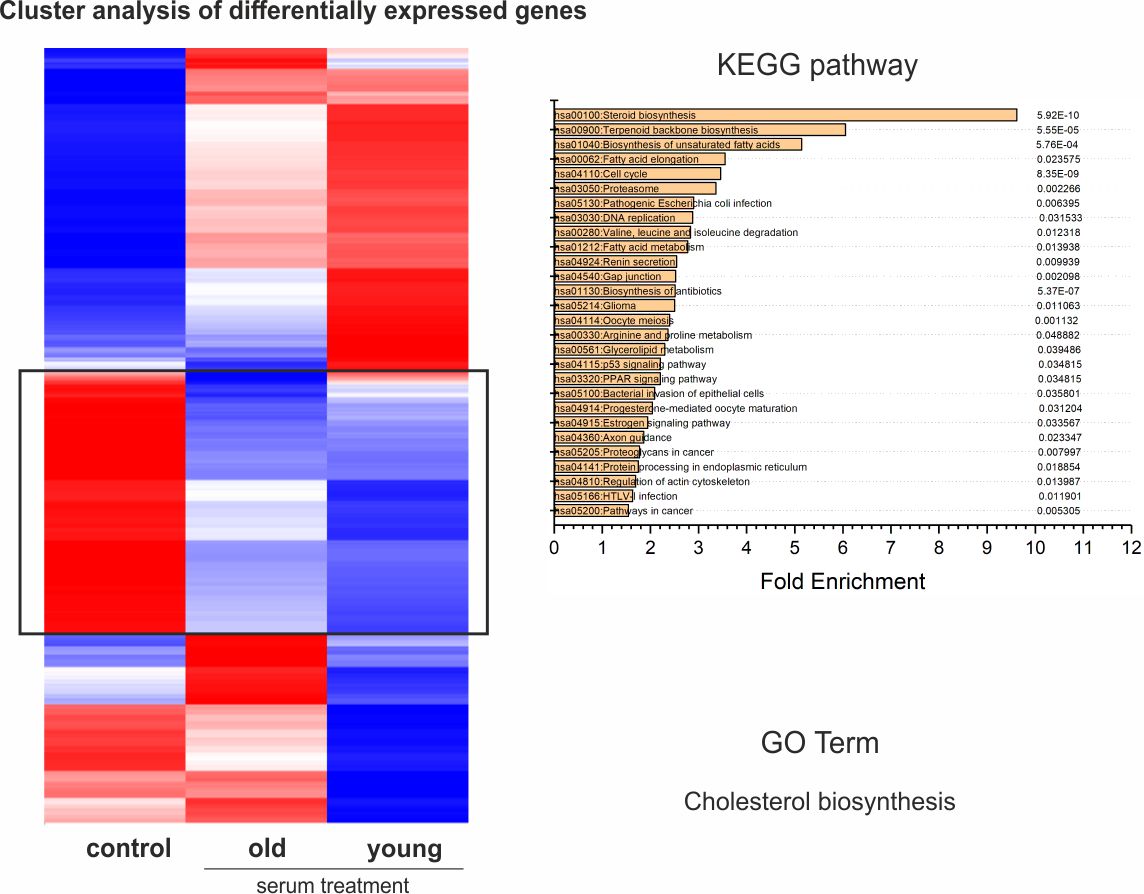

Supplement: Supplementary file 1 [file cells-09-01472-s001.zip › Supplemental material/Figure S3.jpg]

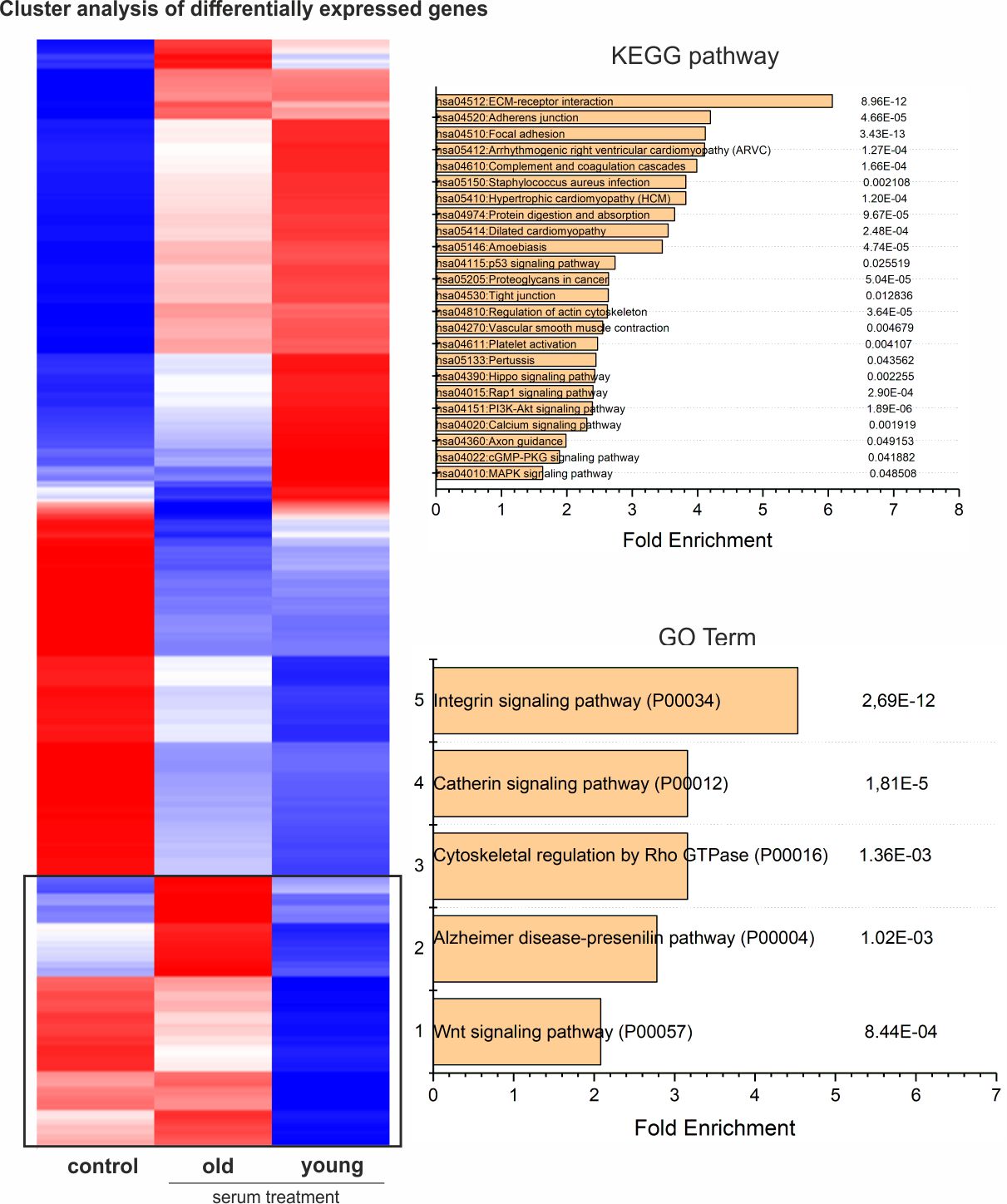

Supplement: Supplementary file 1 [file cells-09-01472-s001.zip › Supplemental material/Figure S4.jpg]
